# Response to selpercatinib in a patient with *RET* fusion-positive pulmonary large-cell neuroendocrine carcinoma: A case report

**DOI:** 10.3389/fonc.2023.1134151

**Published:** 2023-03-14

**Authors:** Aakriti Arora, Jacob Zaemes, Metin Ozdemirli, Chul Kim

**Affiliations:** ^1^ Department of Medicine, MedStar Washington Hospital Center, Washington, DC, United States; ^2^ Lombardi Comprehensive Cancer Center, Georgetown University, Washington, DC, United States; ^3^ Department of Pathology, MedStar Georgetown University Hospital, Washington, DC, United States

**Keywords:** RET fusion, large cell neuroendocrine carcinoma, selpercatinib, lung cancer (LC), targeted

## Abstract

Large-cell neuroendocrine carcinoma (LCNEC) is a rare subtype of non-small-cell lung cancer associated with a poor prognosis. LCNEC is genetically heterogeneous, and studies have revealed distinct molecular subtypes of LCNEC, which may have therapeutic implications. Herein, we present a case of a patient with stage IV LCNEC harboring a *KIF5B*–*RET* fusion whose disease responded to the selective RET inhibitor selpercatinib both extra- and intra-cranially, highlighting the importance of comprehensive molecular testing in LCNEC for selection of optimal treatment.

## Introduction

*RET* fusions are oncogenic drivers found in 1%–2% of non-small cell lung cancer (NSCLC) (1). Patients with *RET* fusion-positive NSCLC are characterized by younger age and never-smoker status and frequently have adenocarcinoma histology (1). Several fusion partners have been identified, including *KIF5B*, *CCDC6*, and *NCOA4* (2). *RET* fusions promote carcinogenesis by activating various downstream signaling pathways such as RAS/MAPK/ERK, PI3K/AKT, and JAK/STAT (2). There are two potent and selective RET tyrosine kinase inhibitors (TKIs) approved by the U.S. Food and Drug Administration: selpercatinib and pralsetinib. Several strategies and new target drugs are currently under investigation (3).

According to the 2021 World Health Organization (WHO) classification of lung tumors, neuroendocrine neoplasms encompass typical carcinoid and atypical carcinoid, as well as high-grade neuroendocrine carcinomas (NECs), including large cell neuroendocrine carcinoma (LCNEC) and small-cell lung cancer (4). LCNEC is a rare subtype of non-small cell lung cancer (NSCLC), accounting for about 3% of all lung malignancies. LCNEC is associated with poor prognosis, with median overall survival less than a year in patients with stage IV disease (5, 6). Although not common, targetable genomic alterations such as alterations to *EGFR*, *BRAF*, and *ALK* are also seen (7, 8).

Here, we describe a patient with stage IV pulmonary LCNEC harboring a *KIF5B*–*RET* fusion whose disease responded to selpercatinib, highlighting the importance of characterizing the molecular profile of pulmonary LCNEC for optimal treatment selection.

## Case presentation

A 52-year-old never-smoking Asian female with no significant past medical history presented with back pain and increasing abdominal girth with firmness in the right upper quadrant. A computed tomography (CT) chest showed a spiculated mass in the right upper lobe measuring 2.1 cm, a left lower lobe nodule measuring 5 mm, extensive mediastinal and hilar adenopathy, diffuse hepatic metastases, and numerous osseous lesions in the thoracic and lumbar spine and in the left iliac bone. A brain magnetic resonance imaging (MRI) scan showed numerous sub-centimeter brain lesions.

**Figure 1:**
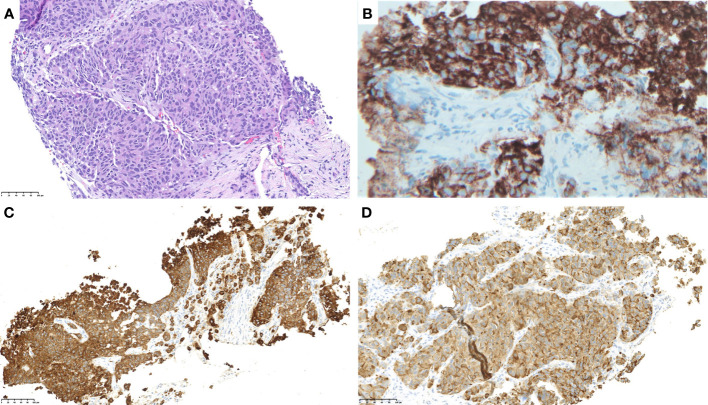
Pathology evaluation of a liver biopsy sample showed a poorly differentiated carcinoma, growing in nests or trabecular patterns without gland formation or keratinization and morphologically resembling large-cell neuroendocrine carcinoma. [**(A)**; ×400 H&E]. The tumor was positive for chromogranin **(B)**, pan-keratin **(C)**, and calcitonin **(D)**.

Subsequently, she underwent a liver biopsy. Pathologic evaluation revealed a poorly differentiated carcinoma, growing in nests or trabecular patterns without gland formation or keratinization and morphologically resembling large-cell neuroendocrine carcinoma (**Figure 1A**). Immunohistochemistry (IHC) was positive for synaptophysin, chromogranin (**Figure 1B**), pankeratin (**Figure 1C**), calcitonin (**Figure 1D**), and CK7 with a high Ki-67 proliferative index of 80%. Other markers, including mammaglobin, GATA-3, CK20, CK903, TTF-1, Napsin A, mucicarmine, WT-1, p63, p16, p53, gastrin, CDX-2, and PAX8, were negative. No lesions were identified on thyroid ultrasound, ruling out the possibility of medullary thyroid carcinoma.

The patient received cisplatin (75 mg/m^2^ on day 1) and etoposide (100 mg/m^2^ on days 1–3), which was complicated by grade 4 neutropenia. Subsequently, molecular testing including whole exome sequencing and whole transcriptome sequencing (Caris Life Sciences, Phoenix, AZ) revealed *KIF5B–RET* fusion and *NFE2L2* E82D without other genomic alterations such as mutations in *TP53* and *Rb1*, and selpercatinib 160 mg twice a day (BID) was initiated on cycle 1, day 21. Ten days after initiation of selpercatinib, she received cycle 2, day 1 carboplatin (AUC 5 on day 1) and etoposide (100 mg/m^2^ on days 1–3). The decision to combine selpercatinib and chemotherapy was based on the limited knowledge about the efficacy of RET inhibitor therapy in LCNEC and the effectiveness of platinum-etoposide for LCNEC, as well as the encouraging initial results from clinical trials testing the combination of platinum doublet chemotherapy and targeted therapy for driver mutation-positive NSCLC, such as EGFR-mutant NSCLC. Cycle 2, day 1 chemotherapy was complicated by reactions including chest tightness and elevated blood pressure during etoposide administration. Chemotherapy was discontinued, and the patient continued selpercatinib at a lower dose of 120 mg BID. A month after initiation of selpercatinib, she developed grade 2 alanine aminotransferase (ALT; 227 units/L) elevation and grade 1 aspartate aminotransferase (AST; 83 units/L) elevation, leading to dose interruption for 10 days. After improvement in liver function tests, selpercatinib was restarted at 80 mg BID, which led to another dose interruption for 10 days due to elevations in grade 2 ALT (239 units/L) and grade 1 AST (95 units/L). Selpercatinib was resumed at 40 mg BID, which was titrated up over 6 months to 120 mg BID without episodes of transaminitis. Due to hypertension, an anti-hypertensive, amlodipine, was started about 5 months after the initiation of selpercatinib.

**Figure 2:**
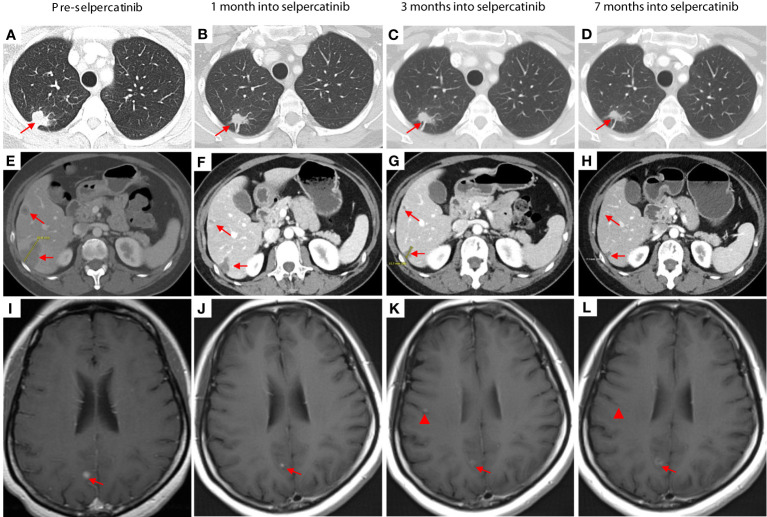
Pretreatment images were obtained 3 weeks prior to the initiation of chemotherapy. The images in the second column were taken 1 month after the start of selpercatinib treatment (3 weeks after the first day of the second cycle of chemotherapy). CT images show shrinkage of lesions in the right upper lobe **(A–D)** and in the liver **(E–H)**. Brain lesions responded to selpercatinib [**(I–L)**, arrows] except for the development of tiny new brain lesions when the patient was on a low dose of selpercatinib 40 mg twice daily [**(K)**, arrowhead], which have improved [**(L)**, arrowhead] or remained stable with subsequent increases in dose of selpercatinib. At 7 months, the patient was receiving a dose of 120 mg of selpercatinib in the morning and 80 mg in the afternoon.

The patient achieved a partial response with shrinkage of the right upper lobe nodule (**Figures 2A–D**) and liver lesions (**Figures 2E–H**). Her brain lesions also responded to selpercatinib (**Figures 2I–L**, arrows), except for the development of tiny new brain lesions when the patient was on a low dose of selpercatinib 40 mg BID (**Figure 2K**, arrowhead), which have improved (**Figure 2L**, arrowhead) or remained stable with subsequent increases in dose of selpercatinib. At the time of this report, the patient is 1 year into treatment with selpercatinib and continues to derive clinical benefit from selpercatinib.

## Discussion

In this case report, we describe a patient with stage IV pulmonary LCNEC harboring a *KIF5B*–*RET* fusion whose disease responded to selpercatinib both extra- and intra-cranially, highlighting the importance of comprehensive molecular testing in LCNEC for selection of optimal treatment. To the best of our knowledge, this is one of the first reports demonstrating the presence of *RET* fusions and the activity of RET inhibitor therapy in pulmonary LCNEC.

LCNEC is associated with a high mutation burden and alterations in various molecular pathways such as cell cycle signaling, RAS/MAPK, and PI3K/AKT/mTOR pathways (9). Studies investigating the molecular characteristics of pulmonary LCNEC have revealed two major subtypes (10, 11): small-cell lung cancer (SCLC)-like LCNEC characterized by co-alterations in *TP53* and *RB1*, and NSCLC-like LCNEC characterized by harboring NSCLC-type mutations. Targetable genomic alterations such as *EGFR* mutations, *KRAS* G12C mutations, *BRAF* V600E mutations, and *ALK* fusions have been identified in LCNEC (8–12), though at lower rates compared with lung adenocarcinoma. Response to matching targeted therapy has been reported for *EGFR* mutations (13, 14), *BRAF* V600E mutation (15), and *ALK* fusions (16, 17), suggesting the role of targeted therapy in driver mutation-positive LCNEC. These findings emphasize the importance of performing comprehensive molecular profiling for patients with LCNEC in order to select the most effective treatment options, in accordance with evidence-based guidelines such as the CAP/IASLC/AMP molecular testing guideline for lung cancer (18).

*RET* fusions have not been well described in pulmonary LCNEC, likely because of the rarity of the genomic alteration and the fact that only a handful of studies utilized genomic technologies involving RNA analysis. In a study of 52 pulmonary LCNECs where reverse transcription-polymerase chain reaction (RT-PCR) was used for analysis, only one patient was found to have a *RET* fusion (12). The patient received first-line treatment with carboplatin and pemetrexed, with progression-free survival (PFS) of 3.3 months and overall survival (OS) of 34 months. No further details about the treatment course are available in the report. There are two case reports describing the activity of selpercatinib against *RET*-fusion-driven high-grade neuroendocrine carcinoma of thoracic origin (19) and atypical lung carcinoid (20), suggesting that, across the spectrum of pulmonary neuroendocrine neoplasms, *RET* fusions are actionable alterations and RET-targeting therapy is a therapeutic option.

Of note, it was observed that the patient developed small new brain lesions when the dose of selpercatinib was decreased due to toxicities. With increased doses of selpercatinib, stabilization and improvement in the lesions were observed. An important clinical question is whether increasing the dose of targeted therapy in cases of central nervous system (CNS) progression is effective. This has been studied in certain driver mutation-positive NSCLCs. For example, in patients with *EGFR*-mutant NSCLC who were experiencing CNS progression while taking osimertinib at 80 mg per day, increasing the dose to 160 mg resulted in modest improvement with CNS control lasting about 3 to 6 months (21). Further studies are necessary to determine the possible advantages of increasing the dose of RET inhibitor therapy for patients with *RET*-fusion-driven NSCLC who are experiencing progression in the CNS.

## Conclusion

Identification of actionable genomic alterations *via* molecular profiling can play an important role in the care of patients with pulmonary LCNEC. RET-TKI therapy is a viable therapeutic option for *RET* fusion-driven pulmonary LCNEC.

## Data availability statement

The datasets presented in this article are not readily available because of ethical/privacy restrictions. Requests to access the datasets should be directed to the corresponding author.

## Ethics statement

Written informed consent was obtained from the individual for the publication of any potentially identifiable images or data included in this article.

## Author contributions

AA: data curation, formal analysis, investigation, visualization, writing—original draft, and writing—review and editing. JZ: data curation and writing—review and editing. MO: data curation, visualization, and writing—review and editing. CK: conceptualization, data curation, investigation, supervision, visualization, writing—original draft, and writing—review and editing.
